# Canonical A-to-I and C-to-U RNA Editing Is Enriched at 3′UTRs and microRNA Target Sites in Multiple Mouse Tissues

**DOI:** 10.1371/journal.pone.0033720

**Published:** 2012-03-20

**Authors:** Tongjun Gu, Frank W. Buaas, Allen K. Simons, Cheryl L. Ackert-Bicknell, Robert E. Braun, Matthew A. Hibbs

**Affiliations:** The Jackson Laboratory, Bar Harbor, Maine, United States of America; The Rockefeller University, United States of America

## Abstract

RNA editing is a process that modifies RNA nucleotides and changes the efficiency and fidelity of the central dogma. Enzymes that catalyze RNA editing are required for life, and defects in RNA editing are associated with many diseases. Recent advances in sequencing have enabled the genome-wide identification of RNA editing sites in mammalian transcriptomes. Here, we demonstrate that canonical RNA editing (A-to-I and C-to-U) occurs in liver, white adipose, and bone tissues of the laboratory mouse, and we show that apparent non-canonical editing (all other possible base substitutions) is an artifact of current high-throughput sequencing technology. Further, we report that high-confidence canonical RNA editing sites can cause non-synonymous amino acid changes and are significantly enriched in 3′ UTRs, specifically at microRNA target sites, suggesting both regulatory and functional consequences for RNA editing.

## Introduction

The commonly taught, simplest version of the central dogma of molecular biology (DNA to RNA to protein) has been complicated in recent years by the discoveries of alternative splicing, non-coding RNAs, and other transcriptional regulatory mechanisms. RNA editing disrupts the faithful transfer of information from DNA to RNA to protein by altering the sequence of RNA molecules co- or post- transcriptionally, potentially altering translational regulation and leading to a diversified proteome. RNA editing was first discovered in 1986 in trypanosomes, where nucleotide insertions cause reading frame shifts [1]. Other forms of RNA editing, including various nucleotide substitutions, deletions, and insertions have been observed in organisms ranging from bacteria to plants to insects to humans [1]–[4]. In mammals, only two classes of RNA editing have been well characterized: cytidine to uridine (C-to-U), and adenosine to inosine (A-to-I). Members of the cytidine deaminase (AID/APOBEC) family of proteins have been shown to catalyze C-to-U reactions on both RNA and DNA substrates [5], [6], however, C-to-U RNA editing is thought to be relatively less common [7]. The majority of known mammalian RNA editing changes are A-to-I, which can be catalyzed by the adenosine deaminase (ADAR) family of proteins [8]. Inosine is read as guanine by reverse transcription and translation machinery, so this type of editing is sometimes referred to as A-to-G editing. Deletion of ADAR proteins is lethal for mice, and dysregulation of A-to-I editing is associated with many diseases, including neurodegenerative disorders, genodermotosis, and cancer [4], [8], [9].

New applications of high-throughput sequencing technology to RNA have expanded the number of characterized A-to-I and C-to-U “canonical” editing sites, and several studies have suggested widespread DNA-RNA differences, including all 10 other possible “non-canonical” base substitutions. However, no enzymes or biochemical processes have been identified that can catalyze these non-canonical RNA edits. Initial computational efforts focused on A-to-I editing and searched through expressed sequence tag (EST) libraries to identify thousands of candidate sites subsequently verified through targeted RNA-seq [10]. Several recent studies utilizing whole-transcriptome RNA-seq have observed both canonical and non-canonical editing sites in human data, including over 10,000 exonic editing events in human B-cells [11], nearly 10,000 sites in a U87MG human glioma cell line [12], and almost 2,000 sites based on paired RNA-seq and DNA-seq of human blood samples [13]. All these studies confirmed a subset of editing sites through Sanger sequencing, and demonstrated that RNA editing can potentially alter coding sequences [11]–[13]. The Li *et al.* study further demonstrated that at least some editing sites are translated into peptides detectable by mass spectrometry [11]. Here, we report that canonical RNA editing occurs in multiple tissues of the important mammalian model organism, the laboratory mouse; however, we report that apparent non-canonical editing is likely an artifact of current high-throughput sequencing technology and analysis limitations.

## Results

**Figure 1:**
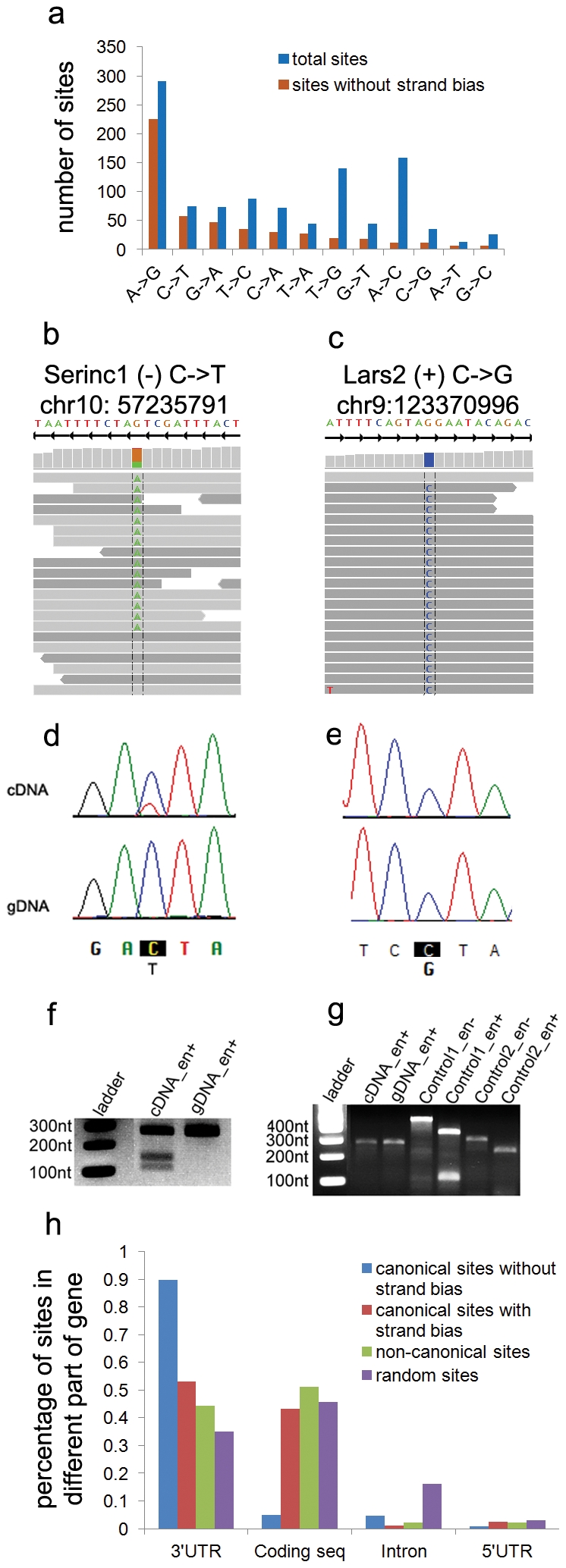
Distribution and validation of editing sites observed in RNA-seq data. A) Distribution of RNA editing types with and without a filter for significant strand bias from RNA-seq data. B,C) RNA-seq traces are shown for one canonical RNA editing site (C-to-U in *Serinc1*) and for one non-canonical editing site (G-to-C in *Lars2*). Reads sequenced in the sense direction are shown in dark grey, while reads sequenced in the reverse direction are shown in light grey. D,E) Sanger sequencing validation results for sites in *Serinc1* and *Lars2*. F,G) RFLP validation of sites in *Serinc1* and *Lars2*. Samples exposed to enzyme are labeled “en+” and control samples are labeled “en-.” H) Genomic distribution of editing sites and random background.

**Figure 2:**
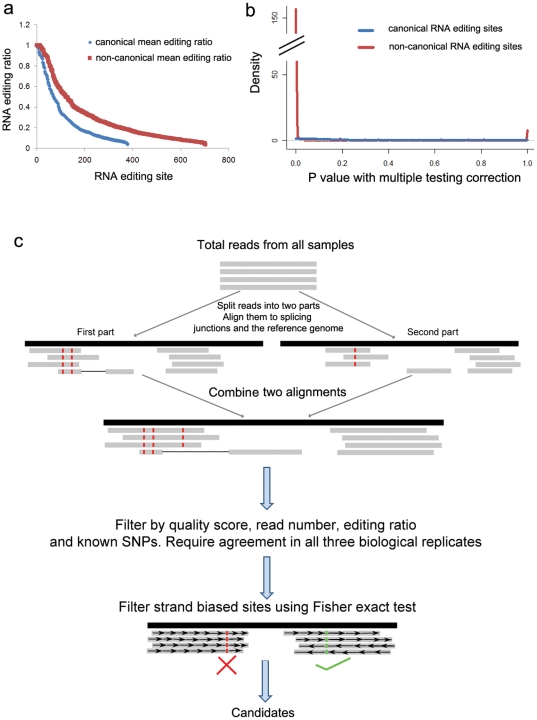
Identification of high-confidence RNA editing sites. A) Distribution of editing ratio for all observed canonical and non-canonical RNA editing sites in our adipose RNA-seq data shows that the ratio is higher for non-canonical than canonical sites. B) Distribution of Fisher's exact test p-values for strand bias. Non-canonical RNA editing sites shows an extreme peak around zero, indicating that most non-canonical RNA editing sites are supported by strand biased reads. C) Overview of our RNA editing analysis pipeline.

We examined RNA editing in the laboratory mouse by sequencing RNA samples extracted from liver, white adipose, and bone tissue from three independent C57BL/6J mice. Data was analyzed by aligning sequenced reads to the mouse reference genome (NCBI build 37; mm9 from http://genome.ucsc.edu) in a manner that tolerates a large number of mismatches and the possibility of reads spanning splice junctions. Potential RNA editing sites were identified as bases supported by at least 2 high quality edited reads with an edit ratio (fraction of edited reads vs. total reads) greater than 5% in all three biological replicates (details in Methods; summary in Table S1). We observed 366 canonical editing sites (A-to-I or C-to-U; Fig. 1A), including 5 A-to-I editing sites orthologous to sites previously validated in human samples (Table S2). Additionally, we observed 683 non-canonical editing sites (all other base substitutions; Fig. 1A), which is consistent with recent reports that all twelve base substitutions are reliably present in RNA-seq [11]–[13]. However, we observed striking differences between the canonical and non-canonical editing sites supported by high-throughput sequencing. The genomic location of canonical editing sites is significantly biased towards 3′ UTRs (p-value<0.001), suggesting a potential function (discussed more below), while non-canonical editing sites are distributed similarly to the genomic background (Fig. 1H). Most significantly, the majority of our non-canonical editing sites (∼70%) are only supported by reads sequenced in a single direction (example in Fig. 1C, summary in Fig. 2B), while the majority of our canonical editing sites are supported by reads sequenced in both directions (Figs. 1B, 2B). This observation is consistent with several recent reports of strand-biased systematic errors present in high-throughput sequencing [14], [15]. The combination of a random genomic distribution and the disparity in strand bias suggests that non-canonical editing may be a result of sequencing errors.

In order to verify that apparent non-canonical editing is an artifact of high-throughput sequencing, we randomly selected 19 canonical and 13 non-canonical editing sites for validation with Sanger sequencing. RNA (reverse transcribed into cDNA) and genomic DNA (gDNA) were extracted from two liver or adipose samples of additional C57BL/6J mice. For each site, the surrounding region was amplified by PCR from both gDNA and cDNA, and each was sequenced. All 19 canonical RNA editing sites were validated in both independent samples through Sanger sequencing (example in Fig. 1D; full results in Fig S1); while none of the 13 non-canonical editing sites were validated (example in Fig. 1E; full results in Fig S2). Further, we validated 1 canonical editing site (C-to-U; chr10:57235791) by a restriction fragment length polymorphism (RFLP) assay utilizing an enzyme (*BspDI*) that cuts only the edited version of the sequence. These results agree with the sequencing results in that the cDNA contains both edited and un-edited forms (since cleaved and un-cleaved products are visible), while the gDNA contains only the un-edited (un-cleaved) sequence (Fig. 1F). Similar RFLP analysis of a non-canonical editing site (G-to-C; chr9:123370996) showed no cleaved products in cDNA or gDNA (Fig. 1G), confirming Sanger sequencing results that the site is not truly edited. Although limited in number, our result of 0 out of 13 non-canonical validations suggests that no more than 3 of our 683 observed non-canonical sites are likely to be genuine, assuming a false discovery rate of 5%. Thus, our results strongly suggest that most canonical editing targets are truly edited, while most non-canonical editing sites identified from high-throughput sequencing are not true editing sites.

Given the large number of observed non-canonical editing sites that show a strand bias in our RNA-seq data and given that systematic high-throughput sequencing biases appear to be quite common [15], it is likely that bias accounts for much of the apparent non-canonical editing observed. However, simple filters are unable to distinguish genuine editing events from these data. For example, the editing ratio is higher for non-canonical editing sites than canonical sites (Figs. 2A, S3). Therefore, we developed a statistical approach to distinguish true editing sites from those caused by sequencing errors by calculating whether edited reads were significantly biased towards one strand using Fisher's exact test. By rejecting editing sites with a Bonferroni corrected p-value<0.05, we eliminate roughly 70% of non-canonical editing sites (including all 13 non-canonical sites that failed to be validated), as well as 30% of canonical sites (Figs. 1A, 2B). After this filtering, the distribution of all types of RNA editing is heavily biased towards canonical A-to-I and C-to-U edits, while the bulk of remaining non-canonical editing sites are complementary to canonical edits, either T-to-C or G-to-A (Fig. 1A). Importantly, two of the best characterized Illumina sequencing errors, A-to-C and T-to-G [15], [16] are almost completely eliminated, suggesting that a strand bias filter may account for most of the RNA-seq specific artifacts. Also consistent with recent reports, using MEME [17], we found a significantly enriched GGC[A/T]GG motif near 55% of strand biased sites (305 of 557, p value = 8.2e-222) (similar to [14]) as well as some enrichment for poly-A sequences (7% of sites, p value = 1.7e-100); and we found a tendency for strand biased sites to be immediately preceded by GG (similar to [15]; all motifs in Fig S6). We did not observe any consistent motifs near sites without a strand bias.

To validate the strand bias filter, we selected 3 canonical editing sites with a significant strand bias for testing (none of the previously validated 19 canonical editing sites displayed a strand bias). None of these sites were validated through Sanger sequencing (Fig S4A), confirming our assessment that strand biased canonical editing sites are likely artifacts. The remaining non-canonical editing sites that do not exhibit a strand bias also appear to be the product of biases or errors, falling into several categories. First, errors or omissions in the reference genome relative to our samples can lead to apparent non-canonical editing. For example, roughly 50% of our observed non-canonical editing sites without any strand bias are localized to a single gene, *Hjurp* (Holiday junction recognition protein; MGI:2685821). Sanger sequencing of several locations within *Hjurp* showed mixed sequencing peaks in both gDNA and cDNA samples (Fig S5), suggesting that there may be additional polymorphic copies of *Hjurp* in our genetic background that are not reflected by the reference genome, leading to observed editing in our RNA-seq data. As such, we removed all sites within *Hjurp* from our final results. A similar phenomenon could occur because of somatic mutations, or genetic drift relative to the reference genome.

Apparent non-canonical editing could also be the result of complementary canonical editing of double stranded substrates or of antisense transcripts. For example, the AID/APOBEC family has been shown to edit C-to-U in DNA sequences, which when repaired can produce a C-to-T edit in the primary strand, and a G-to-A edit in the complementary strand [7]. A similar phenomenon could occur in double stranded RNA substrates. Analogously, the ADAR family of proteins could create A-to-I edits in double stranded substrates that could be repaired to produce A-to-G edits in the primary strand, and corresponding T-to-C edits in the complementary strand. A similar observation could be made if editing occurs in antisense transcripts because of the non-strand specific nature of current RNA-seq techniques. During sequencing library construction, RNA is immediately converted to double stranded cDNA, removing all information regarding strand of origin. As such, we (and others [11], [13]) make the assumption that transcripts originated from the sense strand to identify editing sites. However, recent evidence suggests that antisense transcripts may be broadly produced [18], in which case, canonical editing of antisense transcripts would manifest as complementary non-canonical editing in current RNA-seq data. Interestingly, a recent study examining RNA editing in the presence of siRNA knockdown of the ADAR gene family observed a significant decrease in both A-to-G and T-to-C editing events compared to controls [12]. Complementary editing potentially explains many non-canonical editing sites observed in our data, and may explain other observations of non-canonical editing in the literature.

Due to these potential sequencing errors and biases, we focused further analyses on a set of replicated, canonical RNA editing sites. A summary of our overall approach is shown in Fig. 2C. After applying this RNA-seq data analysis pipeline, including filters for significant strand biases and non-canonical editing, we identified 207 A-to-I editing sites and 35 C-to-U editing sites with high-confidence across all three replicates in at least one tissue (Table S3). These editing sites are significantly biased towards 3′ UTRs (Fig. 1H; hypergeometric p-value<0.001) and against coding regions (Fig. 1H; p-value<0.001). However, the 12 rare canonical coding editing sites are 3 fold enriched for non-synonymous amino acid substitutions, including the 5 sites orthologous to previously validated human editing sites (Table S2), suggesting that RNA editing can diversify the transcriptome and proteome.

**Figure 3:**
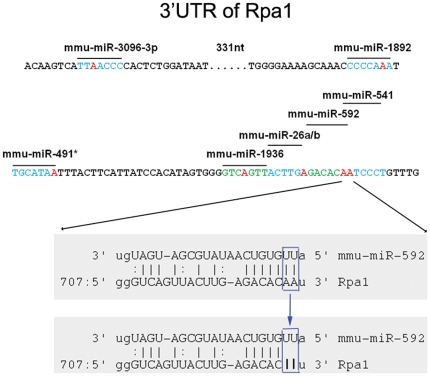
Example of RNA editing at microRNA target sites. One hundred nucleotides of the 3′UTR of *Rpa1* is shown. Multiple microRNA target sites form a dense cluster in this region and contain many A-to-I editing sites. Red bases represent RNA editing sites, and blue and green bases represent different microRNA seed locations.

The majority of our high-confidence RNA editing sites (217 of 242, 90%) occur in 3′ UTRs, and of these, they are significantly biased towards target sites of the predicted “seeds” of microRNAs (2^nd^ to 8^th^ nucleotides of microRNAs, the key positions for microRNAs to recognize their targets [19]) (94 of 217, 43%; p-value<0.001). In contrast, non-canonical sites are not enriched at 3′UTRs and are weakly enriched at microRNA target sites, but given that we have been unable to validate any non-canonical sites, it is unlikely that these edits are genuine. In addition to disrupting known targets, we also found that a significant number of our high-confidence sites potentially create new microRNA target positions (38 of 217, p-value<0.001). Similar results are obtained when using more sophisticated predictions of microRNA targets, such as those from the MicroCosm v5 database [20] (p-value<0.001). This suggests that a primary function of RNA editing could be in the disruption or creation of microRNA targets to affect translational regulation or message stability. For example, in *Rpa1* (replication protein A1; MGI:1915525), a gene essential for replication, recombination, and DNA repair, we observed 12 high-confidence A-to-I editing sites, 7 of which localize to targets of microRNA seeds. Six of these 7 sites are within 100 nucleotides, forming a very dense cluster, including a pair of adjacent editing sites within overlapping targets of microRNAs (Fig. 3). This dense editing cluster could greatly decrease the interaction efficiency between *Rpa1* and microRNAs. This finding is in contrast to previous reports based on computational evaluations of human RNA editing sites, which observed a bias against microRNA target sites, although they did observe rare examples of RNA editing at microRNA target sites [21]. In *C. elegans*, others have shown a genetic interaction between members of the ADAR protein family and members of the RNAi pathway [22], suggesting a role for editing in RNA-mediated interference. Recent efforts have primarily focused on the direct editing of microRNAs and their precursors, rather than on their targets [9], [23]. Our results suggest that RNA editing plays a role throughout the entire process of microRNA-mediated regulation.

## Discussion

We have utilized high-throughput sequencing of RNA followed by rigorous computational analysis to identify a set of 242 very high-confidence canonical RNA editing sites from multiple tissues of the laboratory mouse. Given our experimental and computational results, it is unlikely that non-canonical editing is a true biological phenomenon. For our specific mouse samples, strand bias artifacts account for roughly 70% of observed non-canonical editing sites, reference genome errors account for 15% of artifacts, and the remaining 15% are likely the product of other reference genome imperfections, double stranded substrates, antisense transcripts, or other sources of sequencing error. In addition to the 13 non-canonical strand biased sites that failed to validate, we further examined 1 non-canonical site without a significant strand bias, and it also failed to validate (Fig S4B), suggesting that it may be a result of sequencing errors other than the strand bias issue, and that further analysis of high-throughput sequencing errors is warranted.

Strand bias explains the majority of non-canonical editing sites in our data, while a smaller minority are likely errors or biases from the reference genome; but the balance between these factors may be different for other analysis settings, sequencing platforms, or organisms. The challenge of incomplete or imperfect reference genomes is somewhat mitigated by our use of homozygous C57BL/6J mice, which closely match the NCBI mouse reference genome. However in other populations or organisms, including human datasets, genetic diversity, heterozygosity, and varying qualities of reference genomes will likely present an even greater challenge. In fact, a recent re-analysis of human B-cell transcriptome data [11] demonstrates that the majority of editing sites originally identified may be a product of reference genome discrepancies and resulting alignment errors [24]. However, our results suggest that even in the ideal case of a perfect reference genome and alignments, spurious RNA editing may be observed due to high-throughput sequencing biases. Future studies of RNA editing must account for both complications in alignments to current reference genomes, as well as the biases of sequencing technologies.

Despite mounting evidence from this and other studies, it is difficult to completely rule out the possibility of rare non-canonical RNA editing events, especially for editing sites that are complementary to canonical editing types. Nevertheless, our results suggest that canonical RNA editing plays both regulatory and functional roles, as editing sites are significantly biased towards 3′UTRs and microRNA target sites, and can cause non-synonymous amino acid changes. Although we have some biochemical knowledge of how the AID/APOBEC and ADAR gene families catalyze canonical editing, many open questions remain regarding the regulation and function of RNA editing, including how sites are targeted for editing and which enzymes catalyze which targets. As costs decrease and technologies improve (e.g. single molecule sequencing, strand-specific sequencing, deeper sequencing coverage), future studies of RNA editing will be able to address these outstanding issues.

## Materials and Methods

### Ethics statement

The Institutional ACUC of The Jackson Laboratory approved the use of mice for this study (approval #11003). All animals were treated humanely and in strict accordance with the recommendations provided by the National Institutes of Health.

### Animals

Samples for RNA-seq were obtained from 16 week old female C57BL/6J mice (JR stock #000664), which were purchased from the resource colonies of The Jackson Laboratory (Bar Harbor, ME). Validation experiments were performed in additional, independent C57BL/6J mice, one male and one female. Mice were maintained in polycarbonate boxes (130 cm^2^) on bedding of sterilized white pine shavings under conditions of 12 hours light; 12 hours darkness. All mice had free access to water and rodent chow for the duration of the study.

### Sample preparation and sequencing

RNA was isolated from white adipose, liver, and femurs of three independent animals. RNA-seq was performed using an Illumina GAIIx instrument, and all protocols were followed as recommended by the manufacturer (Illumina, Hayward, CA, USA). Briefly, sequencing libraries were constructed by purifying mRNA from total RNA, which was then fragmented and converted to double stranded cDNA using random primers. Overhangs were converted into phosphorylated blunt ends, and adaptors were ligated to the cDNA fragments. An agarose gel was used to remove excess adaptors and isolate fragments roughly 300 bp long, and PCR was used to enrich for adapter-modified fragments. Libraries were prepared and sequenced using a barcoding scheme across multiple sequencing pools to enable the identification of technical artifacts. As such, each sample was sequenced to a depth of approximately 15 million 68 base pair long paired-end reads (Table S1).

### Validation of RNA editing through Sanger sequencing

RNA and DNA was isolated from livers of two additional, independent C57BL/6J mice. In order to account for potential variation in protocols and reagents, one sample was prepared using a Qiagen kit protocol, and the other using TRIzol (Invitrogen). RNA was digested with DNase I to exclude DNA contamination. Random primers were used for first-strand cDNA synthesis, and Superscript III RT (Invitrogen) was used for reverse transcription. As a control for DNA contamination, DEPC-treated water was used in place of the reverse transcriptase enzyme. PCR was carried out with primers specific for each RNA editing site of interest. Primers were designed to amplify fragments of an appropriate size for sequencing and that could be used on both gDNA, and on potentially spliced cDNA. Amplified PCR products were purified and subjected to Sanger sequencing using standard methodologies. Sites were considered validated if the cDNA sequence at the candidate editing site contained two peaks (both a reference peak and an edited peak), while the corresponding gDNA sequence contained a single reference peak. Sites were considered un-validated if the cDNA and gDNA traces both contained a single reference peak.

### Validation through restriction fragment length polymorphism (RFLP) analysis

We selected two potential editing sites for validation as their sequences were amenable to validation with RFLP analysis. Specifically, the editing site at chr10:57235791 in the *Serinc1* gene creates a sequence that is recognized by the restriction enzyme *BspDI*, and the site at chr9:123370996 in the *Lars2* gene creates an *RsaI* recognition site. In both cases, site specific PCR products for cDNA and gDNA were obtained as described above. These products were subjected to restriction enzyme digest or a mock control for 2 hours at 65°C for *BspDI* and 37°C for *RsaI*. To insure enzyme integrity, two positive controls for *RsaI* were amplified by PCR using specific primers and digested with or without *RsaI*. The resulting fragments were visualized using standard agarose gel electrophoresis.

### Identification of RNA editing sites from RNA-seq data

Our goals for analysis were to identify high-confidence RNA editing sites from inherently noisy high-throughput sequencing of RNA. As such, we analyzed data to filter out poor quality sequences and alignments, but in a manner that tolerates a large number of mismatches in order to account for potential “clusters” of editing sites. First, all reads that do not meet Illumina's chastity filter were removed, and reads that did not contain a perfect barcode sequence were removed, since sequencing errors early in a read are a potential indicator of poor read quality. Second, reads were aligned first to a custom set of all possible exon splice junction sequences based on RefSeq annotations [25], and then to the mouse reference genome (NCBI build 37; mm9 from http://genome.ucsc.edu) by splitting each 68 nucleotide read into two smaller portions which were separately aligned using the Bowtie software [26], tolerating 2 mismatches per portion. Reads were retained for further analysis only if all 4 segments of each paired-end read aligned to the same location with proper orientation and spacing.

Based on these high-quality aligned reads, we identified potential RNA editing sites as genomic locations with mapped reads containing at least 2 high quality mismatched bases (Phred score >20) and where the edit ratio (percentage of mismatched or edited reads out of all reads mapped to that location) is at least 5%. Known SNP locations (from CDGSNPDB 1.3 Mouse [27]) were filtered, as were locations with an editing ratio of 100% across all samples, which are assumed to be SNPs relative to the reference genome, or the result of other errors. In order to filter apparent editing sites due to sequencing strand biases, we calculated a Fisher exact test p-value for each potential editing site covered by at least 20 reads with a 2×2 contingency table of the number of edited forward strand, edited reverse strand, un-edited forward strand, and un-edited reverse strand reads from all reads aligned to the editing site. Editing sites were deemed significantly strand biased and filtered if their Bonferroni corrected p-value<0.05.

### Evaluation of technical variability

Since we were analyzing inherently noisy data for relatively subtle variations, we performed our sequencing using a barcoding scheme that interleaved samples across multiple sequencing lanes and machine runs to enable evaluation of technical variability in sequencing. We applied ANOVA models to evaluate the variability of gene expression levels (measured as RPKM; model RPKM∼run*tissue) and editing ratio (model: EditRatio∼run*tissue). In both cases the run effect did not significantly contribute to variability (effect size <0.05; Fig S7). As such, downstream analyses pooled technical replicates together for analysis.

### Distribution of editing sites and genomic background

The genomic locations of RNA editing sites were classified as 3′UTR, coding, intronic, 5′UTR, or intergenic based on RefSeq gene annotations [25]. In order to determine the genomic background, 10,000 random locations containing at least 20 mapped reads were selected and classified in the same manner. P-values were determined by a 1000 iteration permutation test selecting the same number of random sites and determining if their genomic distribution is more extreme than observed. Similar results are obtained with a coverage requirement of only 2 mapped reads.

### Enrichment of editing sites in “seeds” of microRNA

Editing sites were classified as within a microRNA target site if they fall within the region complementary to the “seed” (2^nd^ to 8^th^ nucleotide) of a microRNA defined by miRBase [28] and identified by our own search through known 3′UTR sequences. Significance p-values were determined by a 1000 iteration permutation test that randomly selects the same number of potential sites (bases covered by at least 20 reads within a sample), and determines how many random samples included as many or more locations at a microRNA target site. In addition to using sequence complementarity to define microRNA targets, we also utilized the targets defined by EBI's MicroCosm v5 database [20], which produced similar results. As before, similar results are obtained with a coverage requirement of only 2 mapped reads.

## Supporting Information

Figure S1 **Canonical RNA editing sites without strand bias validated by Sanger sequencing.** The gene name, coding strand, type of edit, and genomic location are listed for each site. The upper row of bases are oriented relative to the gene's strand, and the lower row of bases are oriented in the direction of Sanger sequencing. Several sites were verified by sequences from both directions.
(TIF)

Figure S2 **Non-canonical RNA editing candidates with a significant strand bias fail to validate through Sanger sequencing.** Results are shown as in Fig S1.
(TIF)

Figure S3 **Distribution of average editing ratios for canonical and non-canonical editing sites.** A) In bone samples, and B) in liver samples. The x-axis is sorted by editing ratio.
(TIF)

Figure S4 **Canonical sites with a strand bias, and a non-canonical site without strand bias fail to validate.** A) Sanger sequencing of 3 canonical sites with a significant strand bias. B) Sanger sequencing of 1 non-canonical site without a significant strand bias.
(TIF)

Figure S5 **Examples of DNA polymorphisms within the** ***Hjurp*** **gene.**
(TIF)

Figure S6 **Motifs discovered at or near strand biased sites.** A,B) Significant motifs within 50 bp of strand biased sites discovered with MEME [17]. C) Distribution of location of motif in A relative to editing site. D) Motif created by aligning all strand biased editing sites at position 51. E-H) Similar to D, but separated by edited base.
(TIF)

Figure S7 **Effect of technical sequencing variation on gene expression and RNA editing.** A) Effect sizes determined from fitting an ANOVA model to gene expression levels (measured as RPKMs) with respect to technical replicate (labeled “run”) and tissue analyzed (model: RPKM∼run*tissue). B) Effect sizes based on ANOVA analysis of edit ratios similarly to A (model: EditRatio∼run*tissue). In both cases, the effect size of run is negligible.
(TIF)

Table S1 Summary of reads and RNA editing sites identified from each tissue.
(DOCX)

Table S2 Homologues of RNA editing sites previously validated in human studies present in our samples.
(DOCX)

Table S3 All high confidence canonical editing sites.
(XLSX)
