# Supplementary figures and images for: Canonical A-to-I and C-to-U RNA Editing Is Enriched at 3′UTRs and microRNA Target Sites in Multiple Mouse Tissues
Source: PLoS One. 2012 Mar 20;7(3):e33720. doi: 10.1371/journal.pone.0033720 (PMC3308996; doi:10.1371/journal.pone.0033720)

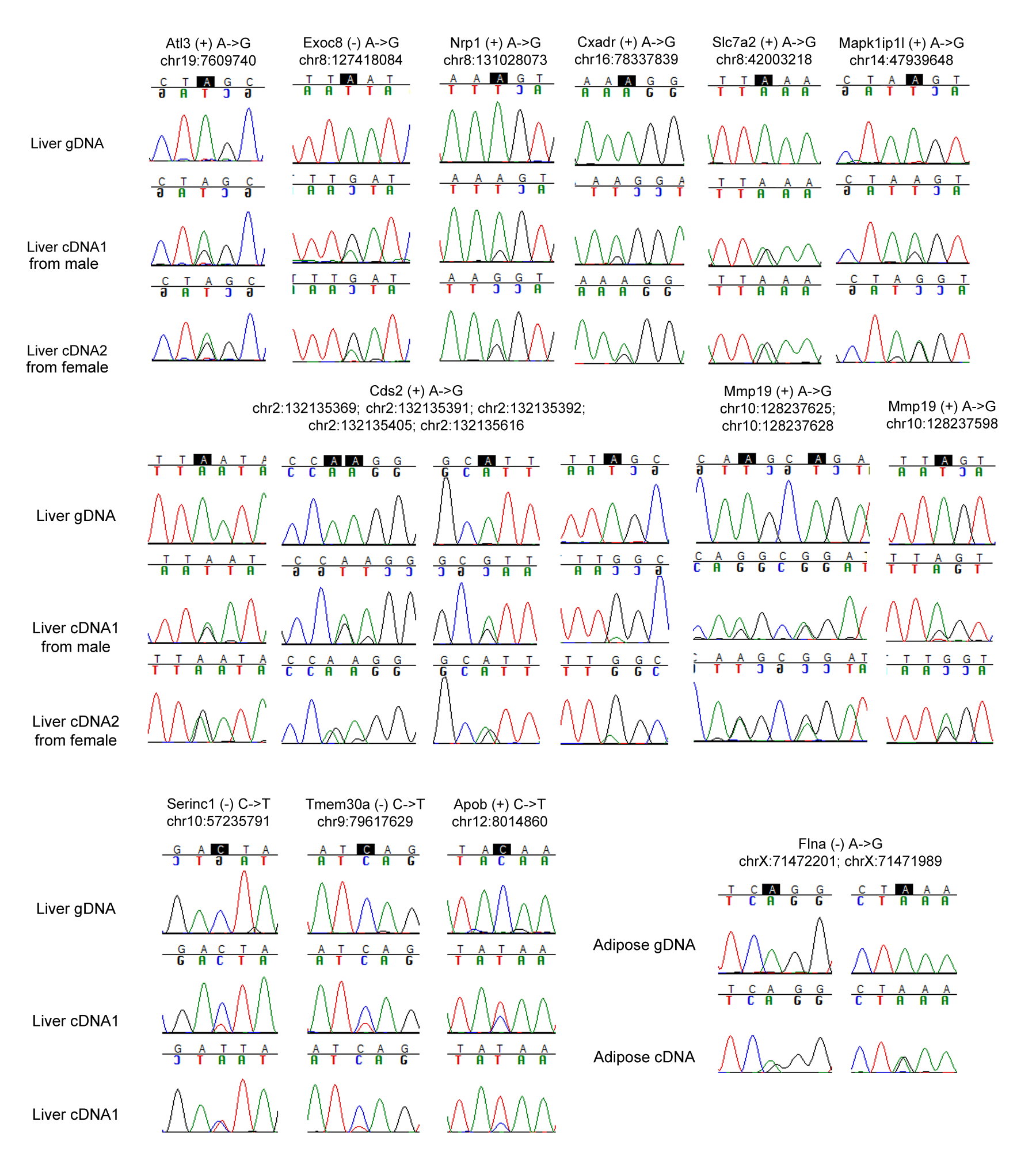

Supplement: Figure S1 — Canonical RNA editing sites without strand bias validated by Sanger sequencing. The gene name, coding strand, type of edit, and genomic location are listed for each site. The upper row of bases are oriented relative to the gene's strand, and the lower row of bases are oriented in the direction of Sanger sequencing. Several sites were verified by sequences from both directions. (TIF) [file pone.0033720.s001.tif]

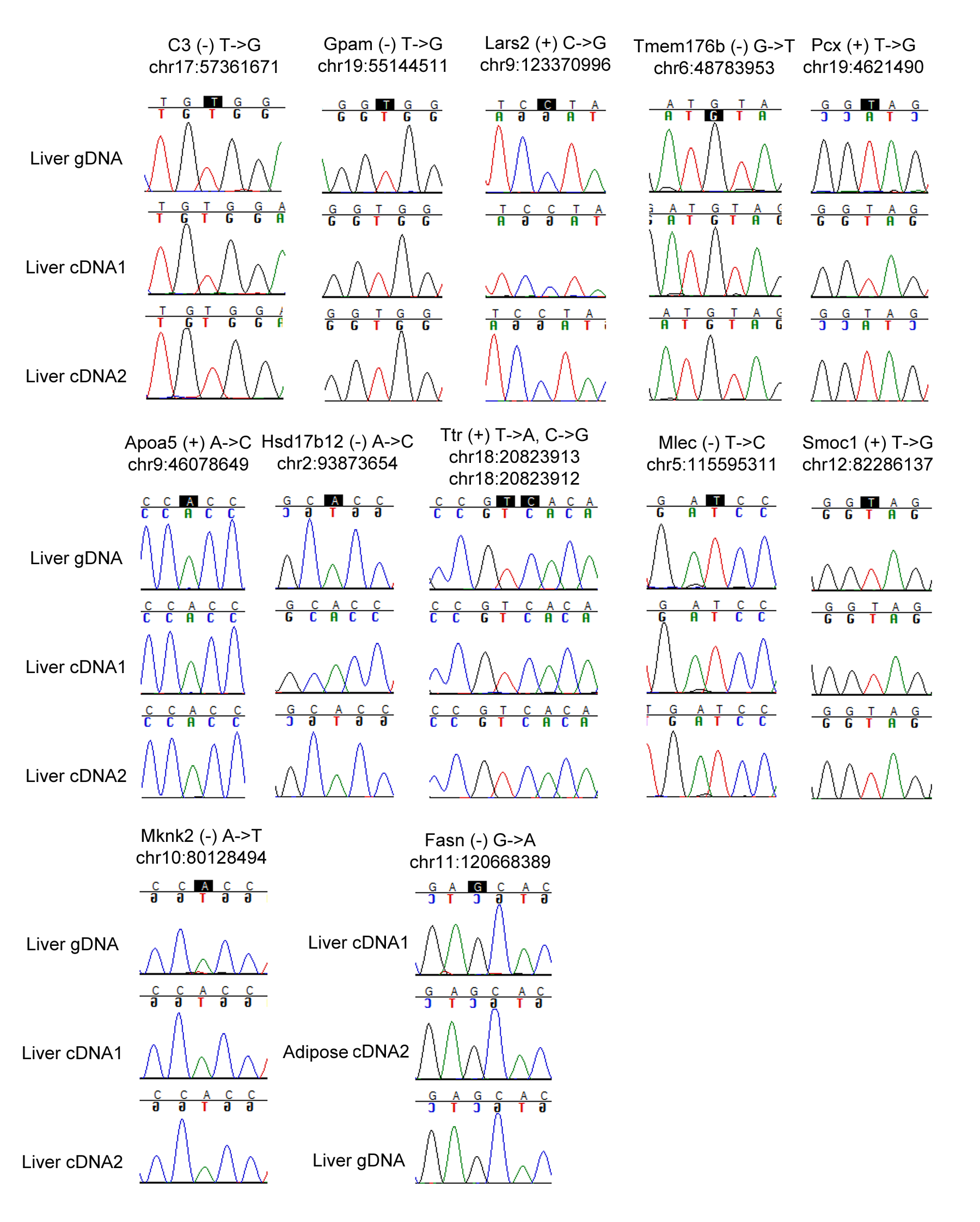

Supplement: Figure S2 — Non-canonical RNA editing candidates with a significant strand bias fail to validate through Sanger sequencing. Results are shown as in Fig S1. (TIF) [file pone.0033720.s002.tif]

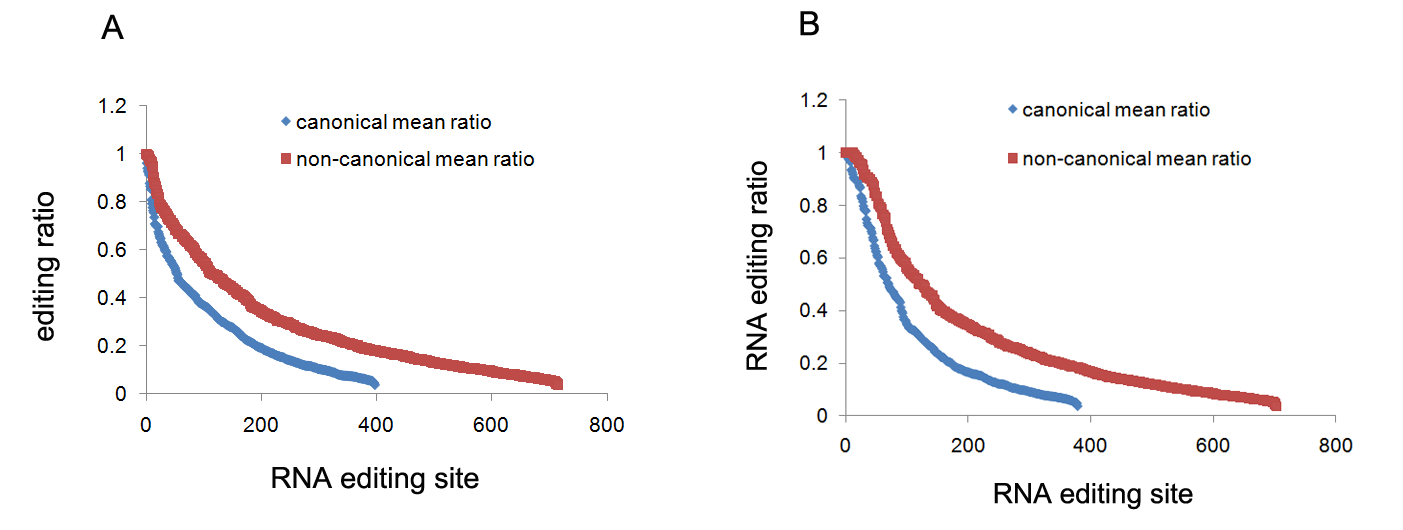

Supplement: Figure S3 — Distribution of average editing ratios for canonical and non-canonical editing sites. A) In bone samples, and B) in liver samples. The x-axis is sorted by editing ratio. (TIF) [file pone.0033720.s003.tif]

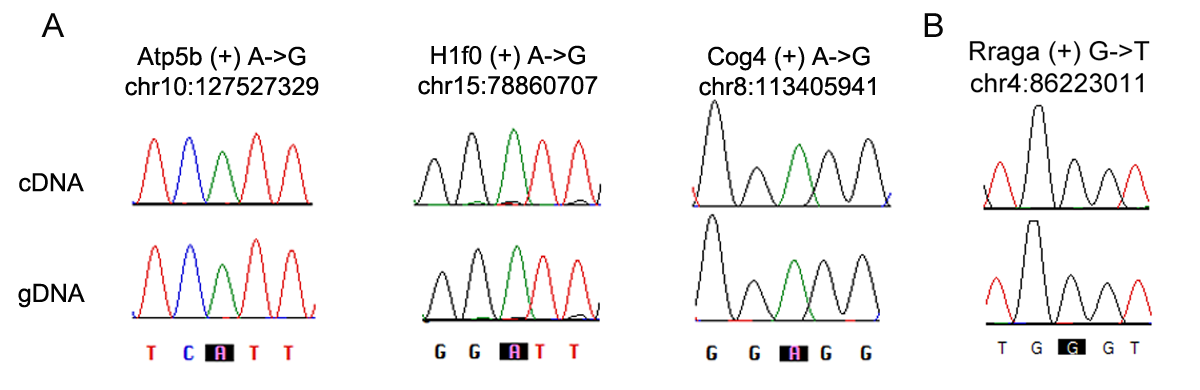

Supplement: Figure S4 — Canonical sites with a strand bias, and a non-canonical site without strand bias fail to validate. A) Sanger sequencing of 3 canonical sites with a significant strand bias. B) Sanger sequencing of 1 non-canonical site without a significant strand bias. (TIF) [file pone.0033720.s004.tif]

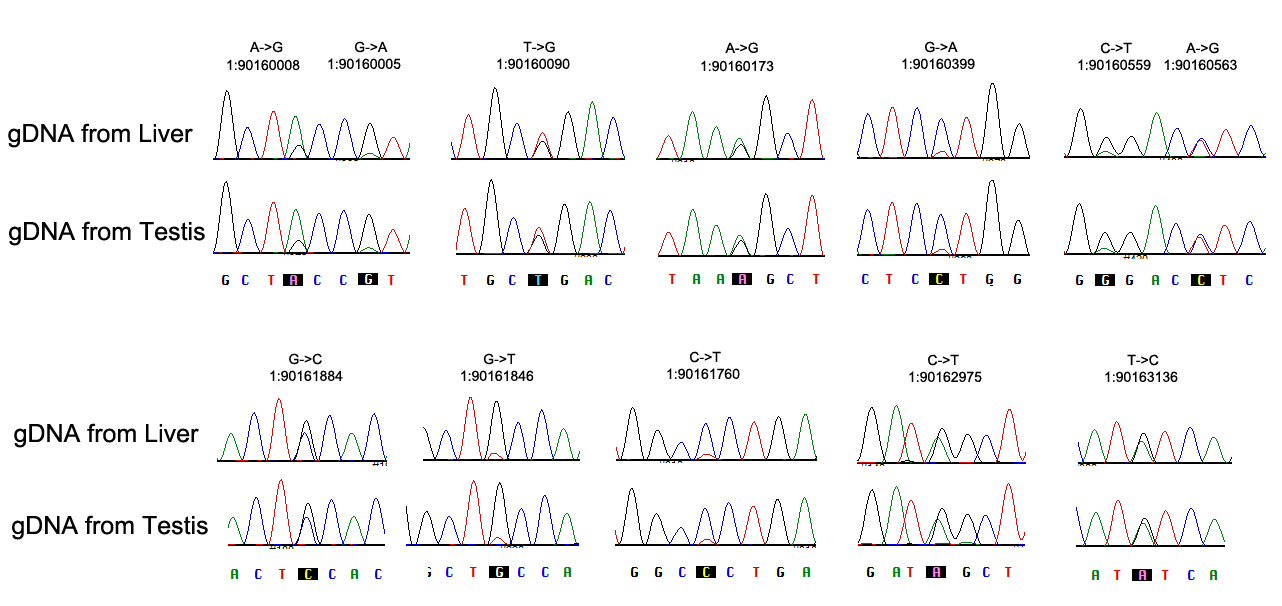

Supplement: Figure S5 — Examples of DNA polymorphisms within the Hjurp gene. (TIF) [file pone.0033720.s005.tif]

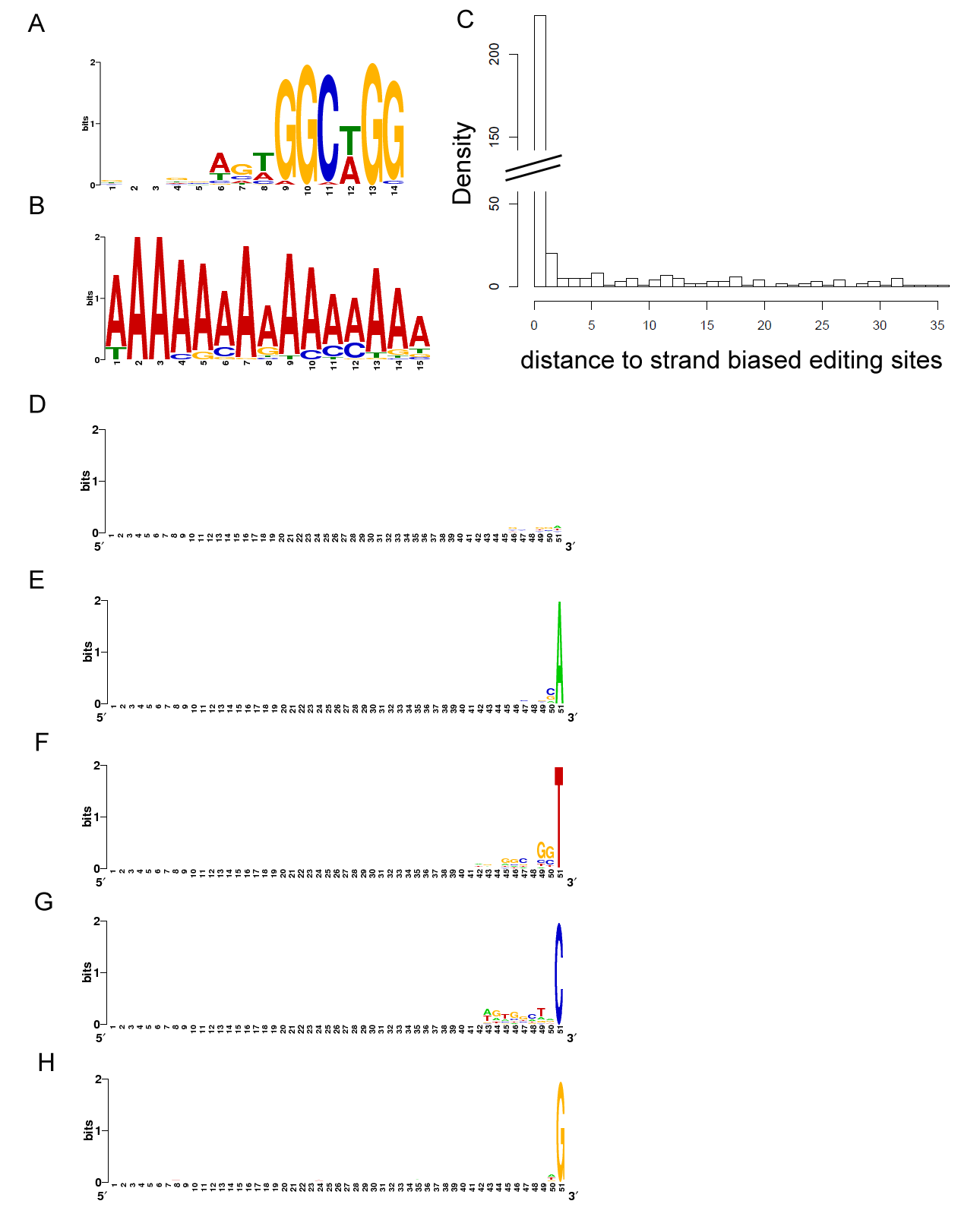

Supplement: Figure S6 — Motifs discovered at or near strand biased sites. A,B) Significant motifs within 50 bp of strand biased sites discovered with MEME [17]. C) Distribution of location of motif in A relative to editing site. D) Motif created by aligning all strand biased editing sites at position 51. E-H) Similar to D, but separated by edited base. (TIF) [file pone.0033720.s006.tif]

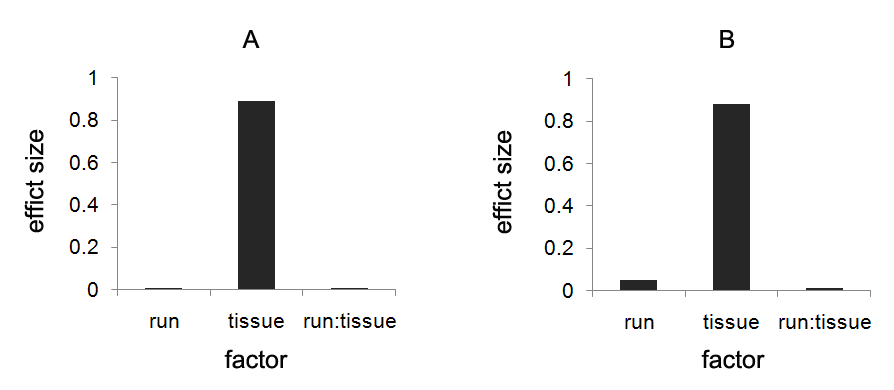

Supplement: Figure S7 — Effect of technical sequencing variation on gene expression and RNA editing. A) Effect sizes determined from fitting an ANOVA model to gene expression levels (measured as RPKMs) with respect to technical replicate (labeled “run”) and tissue analyzed (model: RPKM∼run*tissue). B) Effect sizes based on ANOVA analysis of edit ratios similarly to A (model: EditRatio∼run*tissue). In both cases, the effect size of run is negligible. (TIF) [file pone.0033720.s007.tif]
