# Supplementary material for: Canonical A-to-I and C-to-U RNA Editing Is Enriched at 3′UTRs and microRNA Target Sites in Multiple Mouse Tissues
Source: PLoS One. 2012 Mar 20;7(3):e33720. doi: 10.1371/journal.pone.0033720 (PMC3308996; doi:10.1371/journal.pone.0033720)
Supplement: Table S1 — Summary of reads and RNA editing sites identified from each tissue. (DOCX) [file pone.0033720.s008.docx]

**Table S1. Summary of reads and RNA editing sites identified from each tissue**

| Terms | Adipose | | Bone | | Liver | |
| --- | --- | --- | --- | --- | --- | --- |
|  | Canonical editing | Non-canonical editing | Canonical editing | Non-canonical editing | Canonical editing | Non-canonical editing |
| Avg. reads per replicate* | 23,643,695 | | 25,561,117 | | 21,668,145 | |
| Mapped reads per replicate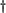 | 11,292,782 | | 11,187,767 | | 8,655,614 | |
| Number of editing sites | 155 | 433 | 216 | 389 | 175 | 381 |
| Number of editing sites without strand bias | 104 | 69 | 164 | 48 | 128 | 63 |

*The average number of total reads from three replicates


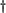
The average number of uniquely mapped reads from three replicates
