# Supplementary material for: Canonical A-to-I and C-to-U RNA Editing Is Enriched at 3′UTRs and microRNA Target Sites in Multiple Mouse Tissues
Source: PLoS One. 2012 Mar 20;7(3):e33720. doi: 10.1371/journal.pone.0033720 (PMC3308996; doi:10.1371/journal.pone.0033720)
Supplement: Table S2 — Homologues of RNA editing sites previously validated in human studies present in our samples. (DOCX) [file pone.0033720.s009.docx]

**Table S2. Homologues of RNA editing sites previously validated in human studies present in our samples.**

| Location | Tissue | Gene | Replicate 1 | | | Replicate 2 | | | Replicate 3 | | |
| --- | --- | --- | --- | --- | --- | --- | --- | --- | --- | --- | --- |
|  |  |  | Mapped reads | Edited Reads | Editing Ratio | Mapped reads | Edited Reads | Editing Ratio | Mapped reads | Edited Reads | Editing Ratio |
| chrX:71472201 | Adipose | Flna | 20 | 14 | 0.7 | 6 | 6 | 1 | 3 | 3 | 1 |
| chr5:77836756 | Adipose | Igfbp7 | 107 | 71 | 0.664 | 143 | 67 | 0.469 | 65 | 31 | 0.477 |
| chr5:77836808 | Adipose | Igfbp7 | 75 | 45 | 0.6 | 58 | 30 | 0.517 | 62 | 20 | 0.323 |
| chr12:8014860 | Liver | Apob | 356 | 244 | 0.685 | 313 | 226 | 0.722 | 241 | 150 | 0.622 |
| chr15:38421367 | Bone | Azin1 | 185 | 21 | 0.114 | 134 | 35 | 0.261 | 143 | 16 | 0.112 |
